# Fabry‐Pérot Microscopy for Improved Contrast Enhancement and Quantitative Phase Imaging

**DOI:** 10.1002/jbio.70217

**Published:** 2026-01-18

**Authors:** Johannes Pittrich, Georg von Köller, Christoph Dillitzer, Daniel Sandner, Ellen Emken, Julia Sistermanns, Zsuzsanna Wolf, Martin Schlegel, Gregor Weirich, Reinhard Kienberger, Oliver Hayden, Hristo Iglev

**Affiliations:** ^1^ Chair for Laser and X‐Ray Physics, School of Natural Sciences Technical University of Munich Garching Germany; ^2^ Heinz‐Nixdorf‐Chair of Biomedical Electronics, TranslaTUM, School of Computation, Information and Technology and Munich Institute of Biomedical Engineering, Technical University of Munich Munich Germany; ^3^ Chair of Methods of Signal Processing, School of Computation, Information and Technology Technical University of Munich Munich Germany; ^4^ German Heart Center TUM University Hospital Munich Germany; ^5^ Department of Anesthesiology and Intensive Care Medicine TUM University Hospital Munich Germany; ^6^ Institute of Pathology Technical University of Munich Munich Germany

## Abstract

Fabry‐Pérot microscopy (FPM) is a label‐free imaging technique that combines a microfluidic optical cavity with a tunable light source to enhance contrast and enable quantitative phase imaging. It integrates a fixed‐length Fabry‐Pérot cavity into a coated microfluidic channel and utilizes resonance‐based interference to selectively highlight structures with specific optical thickness. This design preserves lateral resolution while providing > 20‐fold contrast enhancement compared to conventional wide‐field microscopy. Demonstrations with human oral epithelial cells, blood components, and *E. coli* show clear visualization of subcellular features and differentiation of cell types. Notably, FPM achieves imaging through spectral tuning rather than mechanical scanning, ensuring faster and more stable operation. The system is compatible with standard microscope optics and works under both static and flow conditions, offering significant potential for high‐throughput cytometry, biological research, and in vitro diagnostics. These results establish FPM as a versatile extension of wide‐field microscopy, enabling contrast‐tunable, quantitative imaging of biomedical samples.

## Graphical abstract

Fabry‐Pérot Microscopy (FPM) integrates a tunable laser and on‐chip optical cavity to amplify contrast within biological samples, achieving up to 20× enhancement. This label‐free technique enables quantitative phase imaging and discrimination of different cell types based on their optical thickness.

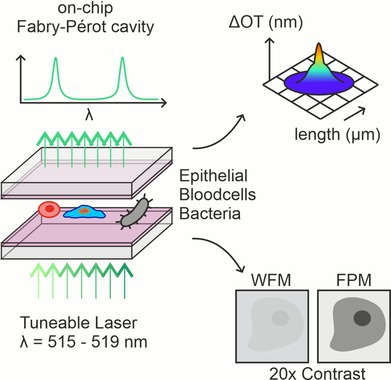

## 1 Introduction

Label‐free optical imaging techniques, such as quantitative phase imaging (QPI) [1, 2] and digital holographic microscopy (DHM) [3], have enabled the quantitative characterization of transparent biological samples without the need for staining. This capability is particularly relevant in biomedical contexts, where subtle changes in cellular morphology or composition serve as key diagnostic markers [1, 4, 5]. For example, the presence of malaria parasites alters the refractive index of erythrocytes [6] but also neurodegenerative diseases like Alzheimer's [7, 8] or diabetes related changes [9, 10] can be characterized. Most of these techniques rely on at least two beams interfering with each other, one reference and one sample beam, and the phase information can be reconstructed using corresponding retrieval algorithms [11]. The emergence of various holographic approaches and their rapid development within the last 20 years [12] led to their increased application in physics, biology, and material science. Nevertheless, these methods often come with increased complexity and high cost, limiting their practical application [13, 14]. Furthermore, there are still systems that exhibit low contrast even for phase‐resolved measurements [11, 15].

An alternative pathway is enabling coherent light to interact with a sample multiple times; this way, the signal‐to‐noise ratio (SNR) can be enhanced, mimicking the benefits typically associated with quantum systems [16, 17, 18, 19]. In this context, multi‐pass microscopy techniques, though conceptually promising, have so far required a lot of optical components and careful alignment [16]. These implementations tend to be bulky, environmentally sensitive, and poorly suited for integration into conventional wide‐field microscopes (WFMs) or quantitative phase microscopes. To overcome these limitations, we present an alternative approach called Fabry‐Pérot Microscopy (FPM), a compact and mechanically stable imaging method that integrates an on‐chip Fabry‐Pérot cavity in a microfluidic sample channel. By plating partially reflective coatings in a microfluidic cuvette, FPM enables light to traverse the sample multiple times, enhancing contrast by nearly an order of magnitude compared to conventional WFM, without compromising spatial resolution. The proposed method allows for background suppression, resonant signal amplification, and quantitative phase extraction while remaining compatible with existing optical microscopy infrastructure. The underlying principle of FPM capitalizes on coherent interference within the optical cavity. As light resonates between reflective coatings, its interaction with the sample is multiplied, accumulating absorption as well as phase shifts and enhancing sensitivity to refractive index variations and optical thickness. By tuning the wavelength of the light to be resonant for the sample, the background will be drastically reduced, resulting in an enhanced signal‐to‐noise ratio. This is perfectly suited for cell recognition and counting tasks like platelet aggregation level measurements that offer prognostic insights [20]. Beyond simple contrast enhancement, FPM enables quantitative phase extraction through continuous tuning of the illumination wavelength. The phase sensitivity depends on the spectral width of the illumination and the properties of the FP resonator (i.e., finesse and free spectral range). By scanning the optical resonance condition, the local optical thickness of the sample can be effectively mapped. In combination with physical thickness measurements, performed using absorption‐based methods with a colored medium, this enables the extraction of intracellular refractive indices with high spatial precision.

In proof‐of‐concept experiments, we demonstrate a height resolution of approximately 40 nm in optical thickness and a Weber contrast enhancement factor of nearly 20× compared to standard WFM. The compact form and low cost of the FPM platform also lend it to automation and high‐throughput applications. For instance, it can be easily integrated into imaging flow cytometry systems, supporting label‐free discrimination and counting of blood cells or bacteria [21]. Furthermore, its operation does not require specialized optical alignment, making it particularly well‐suited for point‐of‐care diagnostics and widespread clinical use. In general, FPM offers a practical, efficient, and scalable solution to the long‐standing challenge of contrast enhancement in biological imaging. Merging principles of multi‐pass interferometry with microfluidic integration opens new pathways for detailed label‐free analysis of cells in both research and clinical settings.

## 2 Experimental Setup and Procedures

### 2.1 Optical Setup and Fabry‐Pérot Cuvette

**FIGURE 1:**
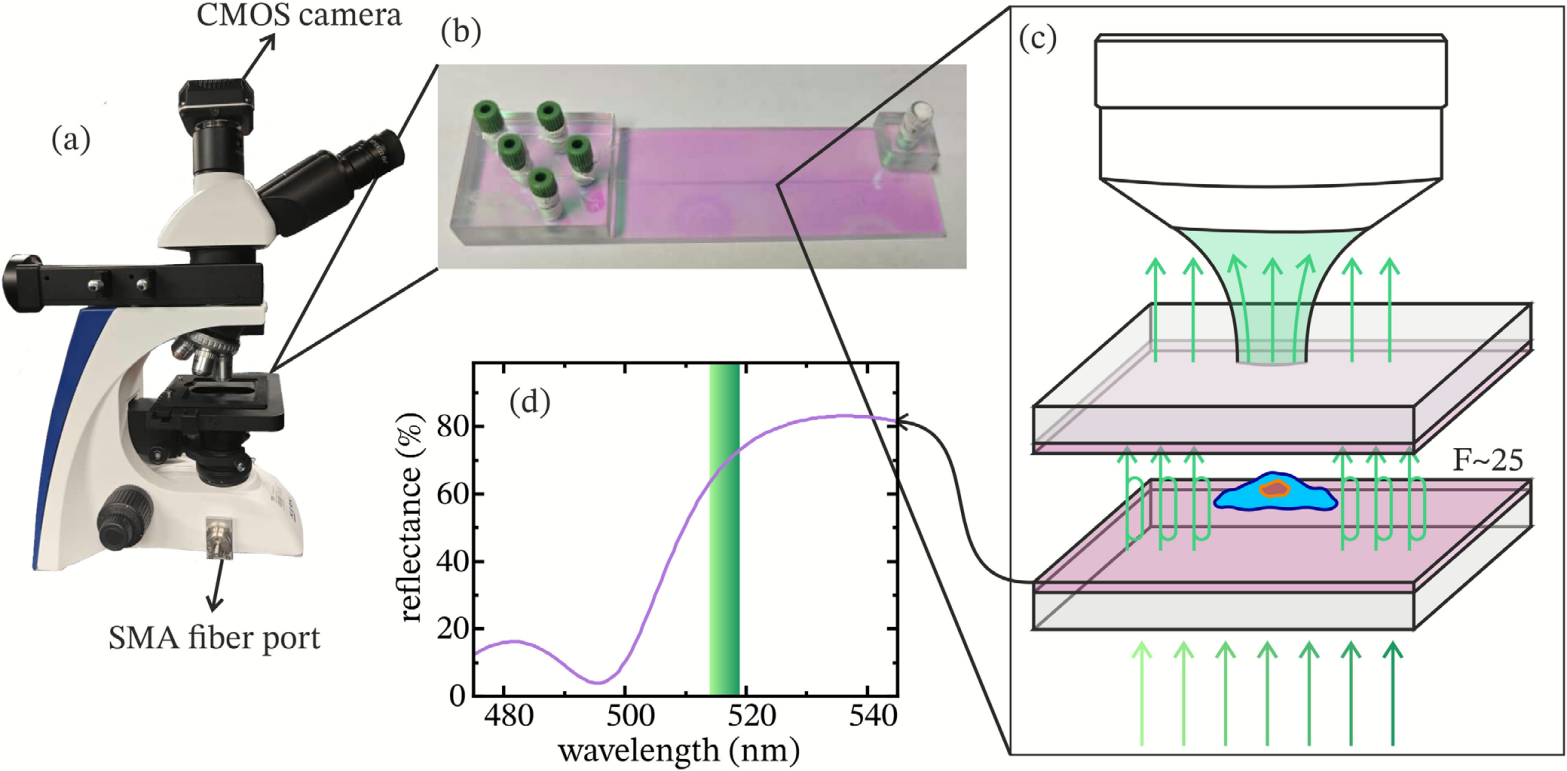
Optical setup and principle of Fabry‐Pérot Microscopy (FPM). (a) Modified wide‐field microscope (KERN OKO‐1) equipped with a CMOS camera and an SMA fiber port for coupling the tunable laser source. (b) Photograph of the microfluidic Fabry‐Pérot cuvette featuring multiple inlet/outlet ports for sample handling. (c) Schematic illustration of light propagation through the Fabry‐Pérot cavity, enhancing contrast by the cavity finesse factor (F *~*25). (d) Measured reflectance spectrum of the cavity coating (purple line), with the tunable laser range (green band).

All imaging experiments presented below were performed using a modified wide‐field microscope (OKO‐1, KERN) adapted for FPM, as shown in Figure 1a. The system was equipped with a 50× objective lens (NA = 0.55) and integrated with a tunable, narrowband laser source (FPYL‐520‐02 T‐TUN, Frankfurt Laser Company) that was fed into the microscope via an additional SMA input connector coupled to an optical fiber. The laser is continuously tunable between 515 and 519 nm, with a spectral bandwidth of approximately 0.29 nm. It operates in continuous wave mode with an output power of 3.81 mW. For better reproducible wavelength tuning, the laser was equipped with a precision stepper motor, which was controlled by our custom‐designed software, as well as a Thorlabs CCS100/M spectrometer to measure the peak wavelength of the light during the measurement.

Light from the tunable laser was coupled into a standard multimode optical fiber and directed into the microscope equipped with collimated illumination and a microfluidic Fabry‐Pérot cuvette. A schematic of the light propagating through the sample is depicted in Figure 1c. The transmitted light was collected by 50× objective and imaged through a tube lens onto a 5.1MP Aptina CMOS (Kern ODC 832), giving a spatial resolution of 0.868 μm/px and a imaging area of 2.25 × 1.69 mm. A custom Python‐based control software automated the acquisition of wavelength‐dependent image stacks. The script set fixed gain and exposure time (usually gain 200 and 350 ms), captured images via the manufacturer's kerncam.dll library, and saved them uncompressed as .bmp files. Simultaneously, it record spectra with the Thorlabs spectrometer using TLCCS_64.dll, storing raw data in .npy format and logging metadata (exposure time, gain, magnification, sample information, and the fitted central wavelength) in a .txt file. The central wavelength of the spectrum is extracted by fitting a Gaussian to the spectrum using the scipy.optimize curve_fit package, with error handling for failed fits.

Between measurement, the wavelength was adjusted mechanically using a NEMA 17 stepper motor coupled to the laser's tuning screw. The stepper motor, driven by an Arduino nano, received steps and direction commands over a serial connection from the Python script. Throughout operation, the script displayed live spectra and images to verify proper data capture.

All measurements were performed under static conditions (no fluid flow), and stacks were acquired across the defined wavelength range. To estimate the effective transmission and finesse of the optical system, the laser's spectrum was cross‐correlated with the theoretical Fabry‐Pérot transmission function. The system's measured finesse factor was primarily limited by the spectral bandwidth of the light source.

The FPM channel shown in Figure 1b consists of an industrially fabricated flow cytometry glass cuvette, monolithically etched, and coated by IMT Masken und Teilungen AG (Switzerland). The cuvette incorporated a microfluidic channel with 50 μm in height, defined during etching. A partially reflective dielectric coating with 60%–80% reflectivity at 515–560 nm was deposited on the inner surfaces of the microfluidic channel, forming a stable Fabry‐Pérot resonator. The cavity supported resonance‐enhanced imaging over the visible spectrum in the 515–560 nm range, where blood cells strongly absorb light [22], with a measured finesse factor of ~25 at 516 nm. A measurement of the reflectance of the coating is depicted in Figure 1d together with the tunable laser range. Adhesive bonding into a monolithic structure provided excellent mechanical stability, eliminating the need for realignment or active cavity control. This robust design allowed reproducible imaging conditions and compatibility with standard microscopy hardware. The microfluidic ports enabled sample loading, but for the experiments reported here, static conditions were used during acquisition. For a supplementary measurement from the high reflecting wavelength range of the coating, the tunable laser was exchanged with a 740 nm laser diode.

### 2.2 Sample Preparation

To evaluate the spatial resolution and axial imaging behavior of the FPM setup, we imaged iron(III) oxide nanoparticles with diameters of 50 nm suspended in water. The size of the nanoparticles is well below the diffraction limit of a standard microscope using a 0.55 NA objective. Human squamous epithelial cells were collected non‐invasively from the inner cheek and suspended in phosphate‐buffered saline (PBS) immediately after extraction. No fixation or staining was applied, and the samples were loaded directly into the Fabry‐Pérot cuvette for imaging under static conditions. To preserve their native morphology and optical properties, all measurements were performed shortly after sample preparation. Peripheral human blood served as the source for both erythrocytes and platelets. Platelets were first isolated by centrifugation and then recombined with red blood cells to generate a mixed sample with approximately balanced concentrations. This suspension, likewise, unstained and unfixed, was introduced into the cuvette and sealed to maintain a static imaging environment. For reference measurements, silica microspheres (SiO₂‐F‐SC31‐4, microparticles GmbH) with a nominal diameter of 7.0 μm (±0.15 μm) were suspended in a mixture of ethanol, deionized water, and an infrared‐absorbing dye. The dye, 1,1′,3,3,3′,3′‐hexamethylindotricarbocyanine iodide (Sigma Aldrich, MKBD8288), exhibits strong absorption at 740 nm while remaining transparent within the FPM spectral window (515–519 nm). This enabled comparative imaging based on absorption contrast. In these measurements, the dye‐filled background appeared dark, whereas silica beads displaced the dye and exhibited increased transmittance.

### 2.3 Data Analysis and Image Processing

The main method of the FPM works by acquiring image stacks where the spectral range of the laser is tuned from 515 to 519 nm in 35 discrete steps. Each frame in the stack corresponded to a specific illumination wavelength measured in parallel with the compact spectrometer. A separate Python script processes the data by loading the previously saved .bmp files and converting them into 2D NumPy arrays of intensity values. These arrays are combined into a 3D dataset representing intensity as a function of position and wavelength *I*(*x,y, λ*). The script then imports the central wavelengths from the metadata file, sorts the images accordingly, and crops the stack to the region containing the relevant sample. A key advantage of FPM is its ability to significantly enhance image contrast by exploiting resonant light transmission. To evaluate this enhancement, we used the absolute Weber contrast:

$$C\left(x,y\right)=\left|\frac{I\left(x,y\right)-I_{b}}{I_{b}}\right|$$

where *I*(*x,y*) is the pixel intensity at a given spatial position and *I*_b_ is the local background intensity. This metric is particularly well‐suited for imaging small biological structures against homogeneous backgrounds, such as single cells in suspension [23]. The absolute Weber contrast was computed for each pixel at each wavelength to generate a stack of contrast images. Then, for the same image position (pixel), the maximum over all the wavelengths was chosen as the respective maximum Weber contrast for each position. With this, one gets the maximum contrast enhancement independent of the optical thickness of the underlying sample, and therefore, it is easy to compare with other microscope techniques, such as WFM.

To extract the quantitative phase, optical thickness maps were generated by identifying, for each pixel, the illumination wavelength at which the transmitted intensity was maximized. The system selectively enhances transmission through sample regions where the optical thickness is a multiple of the laser wavelength, enabling mapping of sub‐cellular structures based on their refractive index and physical geometry. The wavelength with maximal sample transmission, λ_max_, corresponds to the resonance condition of the Fabry‐Pérot cavity and is related to the sample's optical thickness (OT) via OT(x,y) = λ_max_(x,y)/(2 N), where *N* is the order of the interference maximum determined by calibration against background regions. Optical thickness here is defined as the refractive index of the sample (n_s_(x,y)) multiplied with its physical thickness (PT_s_(x,y)) plus the media's refractive index (n_m_) times the remaining length in the cell (d) without the sample OT(x,y) = n_s_…PT_s_(x,y) + n_m_…(d−PT_s_(x,y)). Subtraction of the background reference yielded the relative optical thickness (ΔOT) distribution ΔOT(x,y) = [n_s_(x,y)−n_m_]…PT_s_(x,y).

With a supplementary measurement with a dyed medium, FPM enables quantitative extraction of refractive index values by combining its measurements with physical thickness data. Absorbance measurements in multireflection channels have been done before [24], but to the best of our knowledge, not to extract refractive index information. To demonstrate this, we imaged spherical glass beads suspended in a liquid medium containing the auxiliary dye with selective spectral absorption. The dye was chosen for its minimal absorption at 520 nm, where the FPM operates, and its strong absorption at 740 nm. At the latter wavelength, the dye‐filled medium appears dark, while sample regions displace the dye and appear brighter. By acquiring images at 740 nm, the physical thickness of the glass beads was determined based on local absorbance contrast, using the Beer–Lambert law [25]. First, the absorbance (*A*) of the background media was measured using a UV/Vis spectrometer. With a known channel thickness (*d*_r_) the attenuation coefficient (*μ*) can be calculated (*μ* = *A*/*d*_r_). Now, in the channel, the background intensity (*I*_b_) can be measured as well as the intensity at the sample positions (*I*_s_). With the Beer–Lambert law, we can then arrive at the following equation: PT_s_ = ln(*I*_s_/*I*_b_)/*μ*. To solve for n_s_ we use n_s_(x,y) = n_m_ + ΔOT(x,y)/PT_s_(x,y) to get the refractive indices of the sample at each location.

## 3 Results and Discussion

### 3.1 Preservation of Spatial Resolution

**FIGURE 2:**
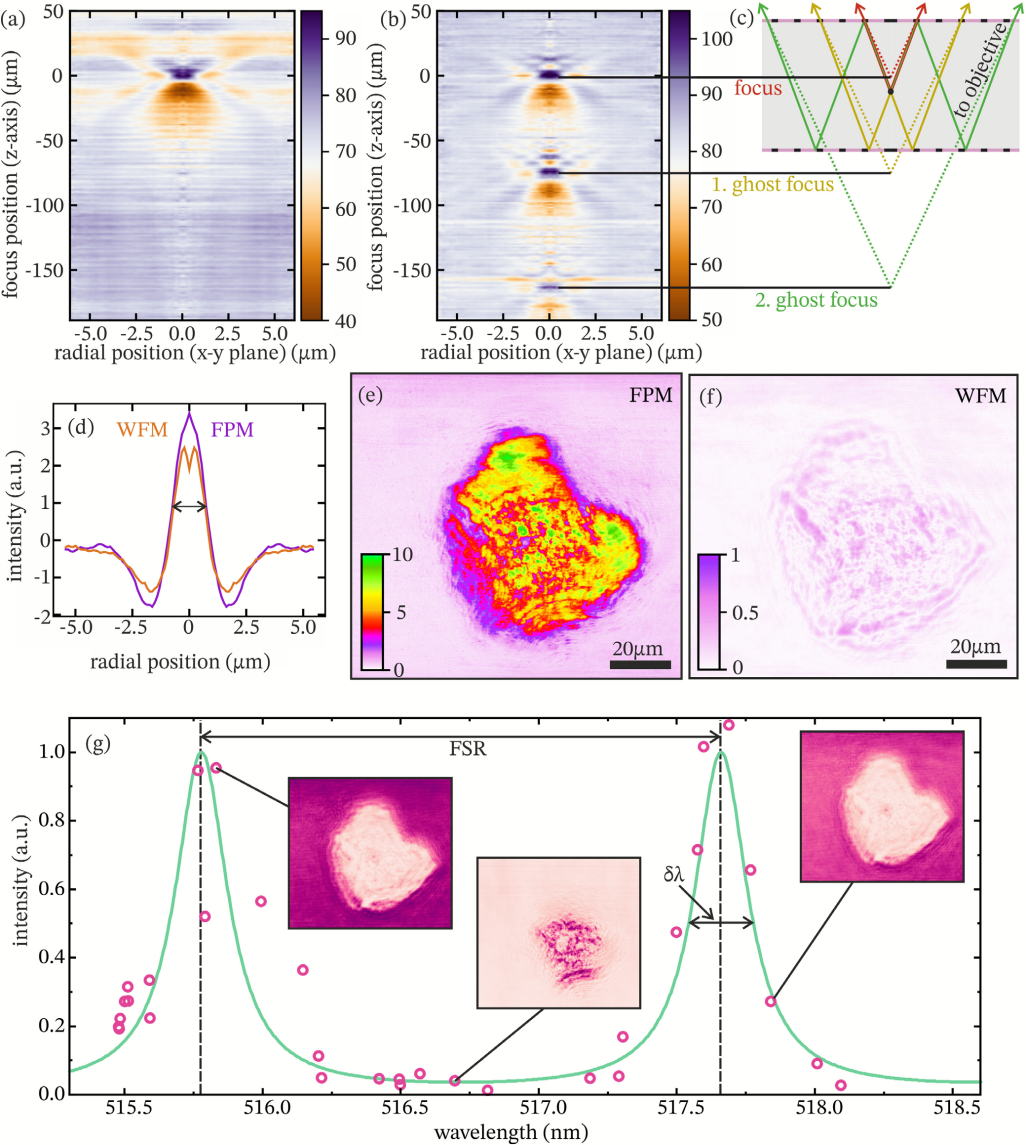
Characterization of FPM. (a) Radially averaged intensity profile around a 50 nm nanoparticle as a function of focus position (z‐axis) for the uncoated cell. (b) Same measurement for the coated FPM cell, showing multiple focal planes due to internal reflections within the cell. (c) Scheme of internal reflections in the coated cell causing multiple nanoparticle images along the z‐axis. Colored rays represent different optical paths contributing to the ghost foci. (d) Point spread function (PSF) of the used microscope in FPM (purple line) and WFM mode (orange), measured with nanoparticles. (e, f) Weber‐contrast image of an epithelial cell measured with FPM and WFM, respectively. (g) Transmission intensity through the cuvette for different illumination wavelengths (experimental points, calculated green line). Insets: Microscope images at corresponding wavelengths.

Characterizing special resolution radial intensity profiles around the nanoparticles were extracted and plotted as a function of axial distance from the focal plane using a Python script, following an approach analogous to Through‐Focus Scanning Optical Microscopy (TSOM) [26]. While the system is not optimized for high‐resolution imaging due to its use of parallel illumination, observing nanoparticles serves as a useful benchmark for assessing whether the introduction of the Fabry‐Pérot cavity degrades the resolution of the microscope. We performed comparative measurements using both uncoated and coated channels. In the uncoated reference channel, the through‐focus scan revealed a single, well‐defined focal plane with a full width at half maximum (FWHM) of 1.21 μm (see Figure 2a,b). In the coated Fabry‐Pérot channel, we observed additional axial features. These ghost images are offset by approximately 73 and 163 μm from the primary focal plane (see Figure 2b,d) and are consistent with internal reflections between the partially reflective cavity surfaces. A schematic of how the ghost foci arise is depicted in Figure 2c. Notably, the reflected images exhibited diminished intensity: the first ghost focus showed a 72% drop relative to the primary peak, and the second a further 65% decrease. These reductions are in line with the expected behavior given the ~68% reflectivity of the cavity coating (Figure 1d). Despite these internal reflections, the main focal peak in the coated channel maintained a FWHM of 1.20 μm (Figure 1d), essentially matching the uncoated channel's resolution within experimental uncertainty.

### 3.2 Contrast Enhancement Through Fabry‐Pérot Resonance

To demonstrate FPM's contrast‐enhancing capability, we imaged an epithelial cell at different illumination wavelengths within the cavity's resonant range. As shown in Figure 2g, the background intensity adjacent to the cell varied strongly with wavelength. These measured background levels (black points) tracked closely with the theoretical transmission spectrum of the Fabry‐Pérot cavity (green curve), confirming that transmission is modulated by the resonant optical thickness of the cavity. Using the Weber contrast as a metric, we first established a baseline by averaging over all wavelengths within one free spectral range resulting in a standard WFM image. The resulting color Weber contrast map is shown (Figure 2f) (with a color code given in the inset) and depicts a maximum value of 0.55 for the WFM operation mode. In contrast, when the same sample was imaged using FPM and wavelength‐tuned illumination, maximum contrast values increased dramatically across the images. The resulting FPM contrast map (Figure 2e) revealed local maxima up to 10.78, representing an approximate 20‐fold improvement compared to WFM. This enhancement closely matches the cavity's finesse factor F ~25. Such interferometrically derived contrast enhancement parallels observations reported for digital holographic and diffraction phase microscopy, where optical path modulation and phase stability lead to significantly improved contrast and SNR compared to conventional wide‐field imaging [27, 28].

### 3.3 Optical Thickness Sensitivity and Quantitative Phase Extraction

**FIGURE 3:**
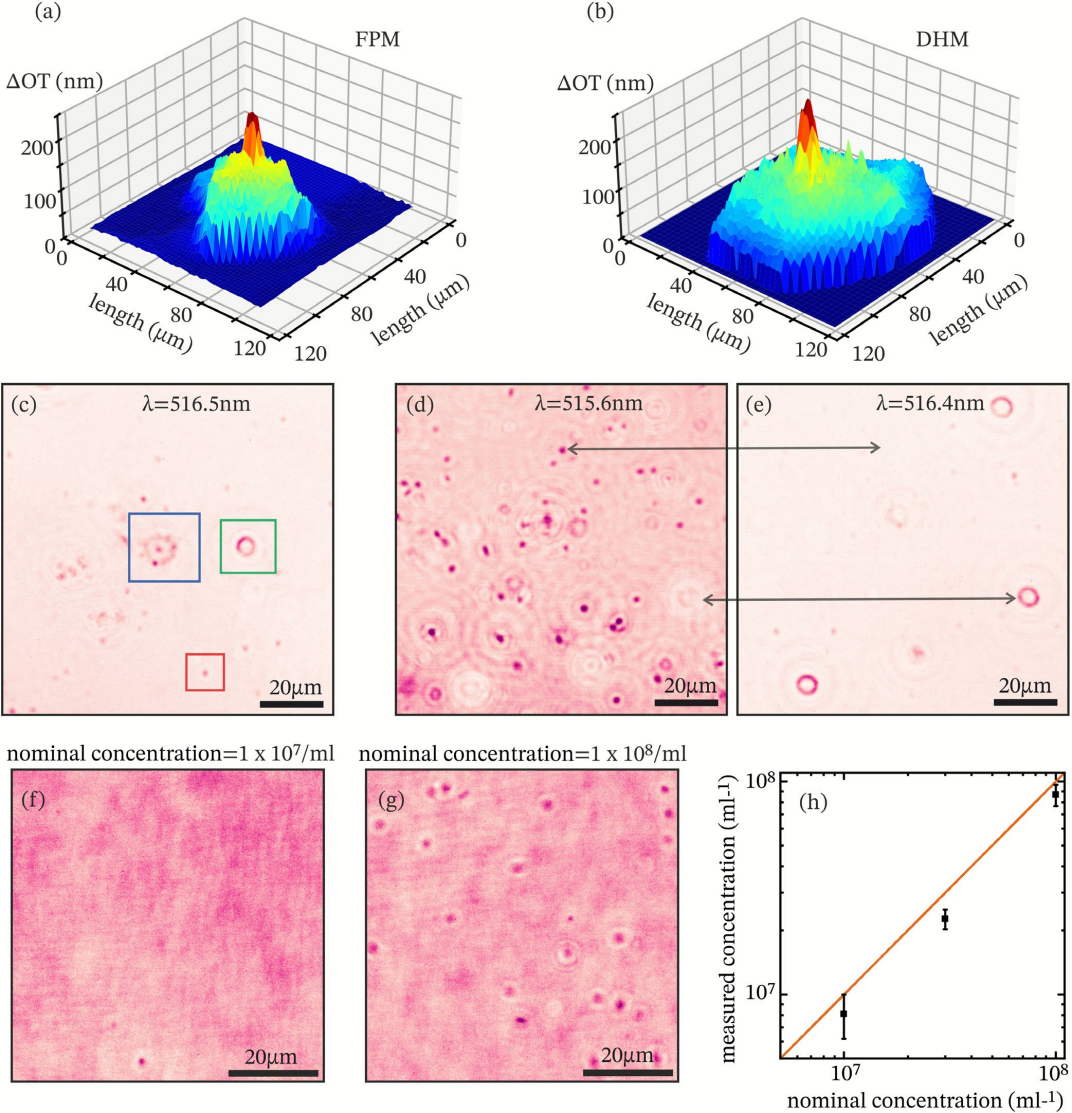
In vitro diagnostic applications of FPM. (a, b) optical path length (OPL) imaging of a human epithelial cell measured with FPM and DHM, respectively. (c) Different types of blood cells: Erythrocytes (green box), immune cells (blue box), and platelets (red box). (d, e) Thickness‐selective imaging of a blood sample at two different wavelengths, highlighting tailored contrast enhancement. (f, g) Bacteria were measured at two nominal concentrations (nc) of 10^7^ (f) and 10^8^ cells/mL (g). (h) Measured versus nominal concentration, while the orange line represents the equality of the two values and serves as a guide to the eye.

Beyond contrast enhancement, FPM is sensitive to optical thickness (OT), which allows quantitative phase information to be extracted from the image data. When applied to epithelial cells, the resulting thickness maps revealed morphological features of the cell body and organelles. Figure 3a shows a reconstructed image from FPM of an epithelial cell. These features were validated by comparison with a digital holographic microscope (DHM, Ovizio Imaging System). The HDM image shown in Figure 3b strongly agrees with the observed topography of the FPM method proposed here. The effective axial resolution of the FPM system was determined by the spectral resolution of the illumination and the order of the interference maximum. This results in a resolution limit of Δ𝑂𝑇_𝑙𝑖𝑚𝑖𝑡_ = 40 nm. By reducing the spectral width of the laser, a resolution limit of around 30 nm could be achieved with the current microfluidic cavity.

### 3.4 Discrimination and Label‐Free Identification of Cell Types

Fabry‐Pérot Microscopy (FPM) enables label‐free differentiation of cell types by using its sensitivity to optical thickness variations. By tuning the illumination wavelength, specific optical thickness values can be selectively enhanced, allowing visual separation of cell populations within mixed biological samples. To demonstrate this capability, blood samples containing erythrocytes, immune cells, and platelets were imaged using FPM. Figure 3c shows a representative field of view, where distinct cell classes were identified based on their morphology and contrast. Erythrocytes (green box), immune cells (blue box), and platelets (red box) each exhibited characteristic optical thicknesses, leading to different resonance conditions at specific illumination wavelengths. This wavelength‐selective enhancement was further illustrated by imaging the same field of view at two different wavelengths (Figure 3d,e). Certain cells appeared prominently at one wavelength but were nearly invisible at another, indicating that their optical thickness matched the Fabry‐Pérot resonance condition only at specific spectral points. This effect confirms that FPM enables selective visualization of cells based on their individual optical properties, facilitating high‐throughput measurement of functional biomarkers. Similar label‐free optical discrimination of blood cell types has previously been demonstrated using DHM, where phase‐based morphological features enabled classification of erythrocytes, leukocytes, and platelets [29, 30, 31].

### 3.5 Quantitative Cell Counting and Detection Sensitivity

Digital holographic microscopy has been successfully applied for label‐free cell enumeration and population monitoring, demonstrating accurate, non‐invasive cell counting in both static and flow configurations [32, 33]. To evaluate the utility of FPM for quantitative diagnostics, we assessed its performance in detecting and counting small biological objects across varying concentrations. Specifically, we measured suspensions of *E. coli* bacteria prepared at three nominal concentrations: 10^7^, 3 × 10^7^, and 10^8^ cells/mL. The stock solution, supplied by TranslaTUM, was concentration‐verified using an Eppendorf OD 600 photometer, then manually diluted, and measured immediately. Imaging was performed under a fixed illumination wavelength using the FPM setup, enabling enhanced detection of low‐contrast bacterial structures. Figure 3f,g show representative FPM images of bacterial suspensions. At both concentrations, bacterial cells appeared as discrete, high‐contrast spots against a homogenous background. For quantitative assessment, bacteria in each field of view were manually counted and converted to concentration by dividing by the observable sample volume (channel height × image area, derived from pixel count and pixel size). Multiple measurements were averaged, and uncertainties were propagated based on the field‐of‐view dimensions. As shown in Figure 3h, measured concentration closely matched the nominal values, yielding a strong linear relationship that approximates an ideal 1:1 correlation.

### 3.6 Refractive Index and Physical Thickness Extraction

**FIGURE 4:**
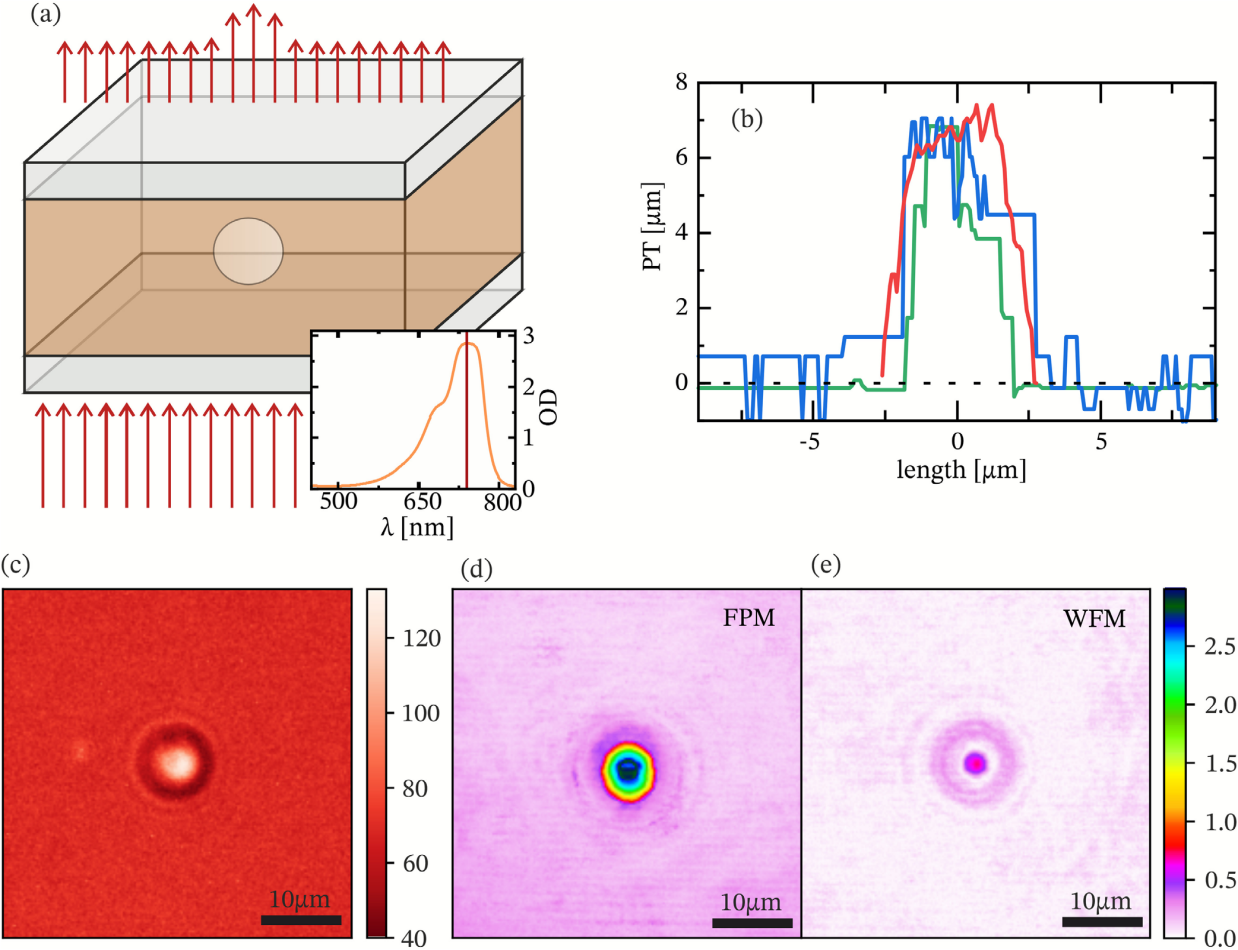
Physical thickness (a) Schematic representation of the setup, where light passes through a sample with an additional dye, enabling physical thickness (PT) measurements shown above. Inset: Absorption spectrum of the added dye, with the selected measurement wavelengths indicated in red. (b) Comparison of physical thickness (PT) profiles of identical glass microspheres obtained through absorption‐based measurements (red line) and FPM performed in suspensions with higher (blue) and lower (green) refractive indices of the solvent. The results demonstrate the feasibility of combining FPM with absorption‐based techniques to determine both the physical thickness and refractive index of the microspheres. (c) Measurement of a micro bead with added dye at 740 nm. (d, e) Weber contrast measurement of a micro bead with the FPM (d) and a corresponding WFM (e).

An additional absorption‐based approach, shown schematically in Figure 4a, enables the physical thickness of the sample to be determined. Figure 4c shows the bright‐field transmission image of the bead recorded at 740 nm illumination, where absorption in the surrounding dye medium provides sufficient background attenuation to resolve physical thickness using the Beer–Lambert law. The extracted diameter of the beads, 6.85 ± 0.2 μm (see the red curve in Figure 4b), corresponds very well with the bead size specified by the manufacturer, 7.0 ± 0.15 μm. Using this thickness along with the optical thickness values extracted from FPM measurements, the resulting refractive index of the glass beads was 1.45 ± 0.01 (Figure 4b, green and blue curves), consistent with known values for the material. These results highlight the potential of FPM as a point‐of‐care method for rapid analysis of bacterial infections and antimicrobial resistance. To further illustrate the contrast enhancement provided by Fabry‐Pérot microscopy, Figure 4d,e compare the corresponding Weber contrast maps for FPM and WFM of a single microsphere. The maximum Weber contrast reached 2.99 for FPM (see Figure 4d) and 0.72 for WFM (Figure 4e), corresponding to a 4.15‐fold improvement under otherwise identical imaging conditions. Although the contrast enhancement is less pronounced than for the epithelial cell measurements, this difference can be attributed to lensing effects within the spherical microbeads that introduce local distortions in the transmitted intensity. Nevertheless, achieving more than a fourfold improvement in contrast under these challenging conditions further demonstrates the robustness and versatility of FPM for quantitative imaging.

## 4 Conclusions

The proposed method, referred to here as Fabry‐Pérot microscopy (FPM), integrates a fixed‐length Fabry‐Pérot cavity into a reflection‐coated microfluidic channel. Multiple beam interference between these partially reflective mirrors results in resonant amplification of transmitted light at specific optical path lengths, while suppressing the signal for the remaining sample thicknesses. This makes FPM highly sensitive to changes in wavelength of the narrow‐band illumination, refractive index, or sample thickness, which shifts the resonance condition. This provides a powerful method to visualize low‐contrast, transparent objects that typically remain undetectable in standard wide‐field microscopy (WFM). We demonstrated that FPM improves more than 20‐fold the image contrast without compromising lateral resolution using a scalable consumable solution for diagnostics. Different cell types or structures, which exhibit small but distinct variations in refractive index and thickness, resonate at different wavelengths. As shown in the discrimination of blood components, this property can be used to highlight specific cell types without chemical labels. The ability to distinguish unlabeled platelets, leukocytes, and erythrocytes in a complex sample represents a significant advancement for label‐free imaging cytometry.

The technique also enables quantitative phase reconstruction by analyzing wavelength‐dependent changes in intensity. Unlike classical tomographic approaches, which require mechanical scanning or complex interferometry, FPM uses the spectral information to extract depth information from 2D image stacks. This enables reconstruction of nanometer‐scale optical thickness profiles in the nanometer range from a compact, stable arrangement. The on‐chip integration of the Fabry‐Pérot cavity within a microfluidic cuvette offers superior mechanical stability, enabling a versatile imaging method for biomedical research and diagnostics, with potential for applications ranging from in vitro cell analysis to portable point‐of‐care diagnostic systems.

While FPM offers powerful imaging capabilities, it also has several limitations that require improvement. A key challenge is phase wrapping in optical thickness measurements when the sample's optical thickness exceeds half the illumination wavelength. This can be mitigated through dual‐wavelength imaging or broad spectral tuning. A narrow‐band laser combined with a higher mirror reflectivity of the cavity could significantly enhance the axial resolution and contrast enhancement beyond the currently achieved values. Additionally, there is strong potential for combining FPM with other imaging modalities. Combining FPM with fluorescence microscopy or Raman microscopy could merge structural and molecular specificity. Its resonance sensitivity to refractive index changes may enable monitoring of cellular processes such as apoptosis, activation, or osmotic shifts [34, 35].

## Conflicts of Interest

The authors declare no conflicts of interest.

## Data Availability

The data that support the findings of this study are available from the corresponding author upon reasonable request.
